# High-resolution images in macular disorders

**DOI:** 10.22336/rjo.2021.41

**Published:** 2021

**Authors:** Radu Ochinciuc, Uliana Ochinciuc, George Baltă, Leila Al Barri, Cristina Pac, Teodoru Adrian, Florian Baltă, Marian Burcea

**Affiliations:** * Department of Ophthalmology, “Victor Babeș” University of Medicine and Pharmacy, Timișoara, Romania; ** Department of Ophthalmology, “Dr. Carol Davila” Central Military Emergency University Hospital, Bucharest, Romania; *** Emergency Eye Hospital and Clinic, Bucharest, Romania; **** Department of Ophthalmology, Faculty of Medicine, “Lucian Blaga” University of Sibiu, Romania; ***** Department of Ophthalmology, “Carol Davila” University of Medicine and Pharmacy, Bucharest, Romania

**Keywords:** adaptive optics, fundus autofluorescence, cone mosaic, cone density

## Abstract

**Objective:** This study analyzed and compared the results of adaptive optics (AO) and fundus autofluorescence (FAF) in various maculopathies.

**Methods:** The study included four different types of maculopathy: central serous chorioretinopathy (CSC), retinitis pigmentosa (RP), Stargardt disease (STGD) and phototoxic retinopathy. In all four cases, cone mosaic and cone density were obtained using AO fundus camera. Further, the high-resolution images were compared with the FAF and optical coherence tomography (OCT) results.

**Results:** In CSC, FAF and AO, changes could be shown in the macula even two years after the subretinal fluid resorption, as opposed to a normal OCT. The improvement of FAF and cone mosaic appearance was concomitant with the visual acuity growth. Several cone mosaic phenotypes were observed in RP and STGD. In RP, the cone density was 24.240 cones /mm2 in the center, and decreased to 8.163 cones/ mm2 in the parafoveal area. In STGD, the cone density was lower in the center, 9.219 cones/ mm2, and higher at the periphery, 12.594 cones/ mm2. In the case of phototoxic retinopathy, AO and OCT were more effective than FAF in highlighting the photoreceptor and retinal pigment epithelium lesions.

**Conclusions:** FAF and AO are very useful tools in macular pathologies examination. FAF can offer a true picture of the metabolic changes in the macula, while AO allows the view of changes up to the cellular level.

**Abbreviations:** STGD = Stargardt disease, CSC = central serous chorioretinopathy, RP = retinitis pigmentosa, AO = adaptive optics, FAF = fundus autofluorescence

## Introduction

Macular disorders are an important cause of vision loss in adults and young people as well [**1**]. Age-related macular degeneration is by far the most common cause in the elderly, followed by vascular disorders such as diabetic maculopathy and vascular obstruction [**2**,**3**]. Central serous chorioretinopathy (CSC) and retinal dystrophies are more common under 40 years of age [**4**-**6**].

Adaptive optics (AO) is a new imaging technique that allows the acquiring of high-resolution images of the retinal vessels and the outer layers of the retina [**7**]. The analysis of the outer layers of the retina offers information about the dispersion and density of the photoreceptors and about the size of the intercellular spaces. 

Fundus autofluorescence (FAF) is also a noninvasive technique that allows the quickly highlighting of the lesions in the retinal pigment epithelium (RPE) and in the photoreceptors layer. Any functional or anatomical disorders of these two structures lead to changes in the amount of autofluorescent material, which will cause hypo- or hyper-autofluorescent lesions.

The purpose of this paper was to highlight and compare the lesions of certain macular pathologies with the help of AO and FAF. These results might provide additional information about the pathophysiological mechanism or how to approach these patients in the future.

## Methods

This study received approval from the Institutional Review Board and the Ethics Committee of “Ponderas” Academic Hospital. All procedures conformed to the tenets of the World Medical Association’s Declaration of Helsinki. All patients included in the study signed the written informed consent.

This article described a series of four cases with different maculopathies as it follows: central serous chorioretinopathy (CSC), retinitis pigmentosa (RP), Stargardt disease (STGD), and phototoxic retinopathy. Following the functional and clinical ophthalmological examination, optical coherence tomography (OCT), FAF and high-resolution images using AO were performed in all patients for diagnostic and prognostic purposes.

For high-resolution images attainment, the AO Retinal Camera rtx1TM (Imagine Eyes, France) was used. The AOimageTM software (Imagine Eyes, France) allowed the correction of the refractive error and the setting of 70 μm depth of focus in all patients. After making nine images that covered an area of 8° × 8° from the macula, the AO i2k retinaTM software (Imagine Eyes, France) was used to obtain the cone mosaic. Photoreceptor density was determined automatically with the AOdetect mosaicTM software (Imagine Eyes, France). 

FAF and OCT were performed using Swept Source DRI OCT Triton plus (TOPCON, Japan). The 6-mm radial scans of the macula were obtained in all patients. Fluorescein angiography (FA) was performed in the patient with CSC for diagnostic purposes and to guide the LASER treatment. 

## Results

*Case 1*

The first case was a 40-year-old man with focal laser treated CSC in his right eye (RE). Best corrected visual acuity (BCVA) at the presentation was 0.4 (decimal fraction), which has improved to 0.8 in one year and 1.0 in two years. Before treatment, the patient presented:

- ophthalmoscopy: a round serous macular detachment with small yellow sub-retinal deposits;

- OCT: neurosensory detachment, elongation of the photoreceptors outer segment and hyperreflective dots in the subretinal fluid (SRF);

- FA: point leak within the macula in early phase, that gradually increased in intensity and size in midphase;

- FAF: the edges of neurosensory detachment slightly hypoautofluorescent (hypo-AF), hyperautofluorescent (hyper-AF) dots and three hypo-AF lesions.

**Fig. 1:**
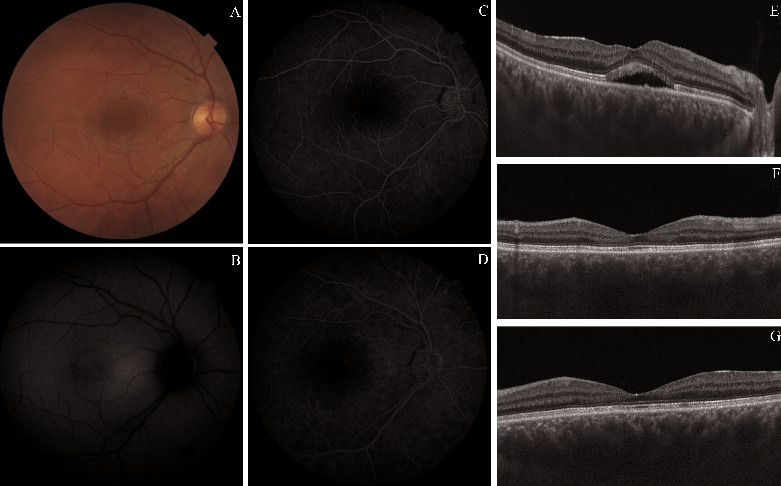
Central serous chorioretinopathy (CSC). **A.** Color fundus image. **B.** Fundus autofluorescence image. **C, D.** Fluorescein angiography - early phase and midphase. **E, G.** Optical coherence tomography scan during active CSC **(E)**, at one **(F)** and at two years **(G)** after the focal LASER photocoagulation

After comparing the FA with FAF images, it was observed that the leakage point overlapped with a hypo-AF lesion (**Fig. 1**). 

The SRF disappeared in about two months. In this paper, we compared AO and FAF images taken one and two years after the treatment. The most important black (hyporeflective) and white (hyperreflective) lesions were noted on the AO images. No differences were observed between one and two-years-old OCT.

One year after the focal LASER photocoagulation:

- FAF: the edges of neurosensory detachment still visible but less hypo-AF; less hyper-AF dots and five hypo-AF lesions (including the photocoagulation point);

- AO cone mosaic: blurred cone mosaic in the fovea, with intensely white and black lesions; normal and blurred mosaic in the parafoveal area.

Two years after the focal LASER photocoagulation:

- FAF: the edges of neurosensory detachment less hypo-AF, less hyper-AF dots and four hypo-AF lesions;

- AO cone mosaic: normal and blurred mosaic alternate, with less intensely white and black lesions in the fovea.

**Fig. 2:**
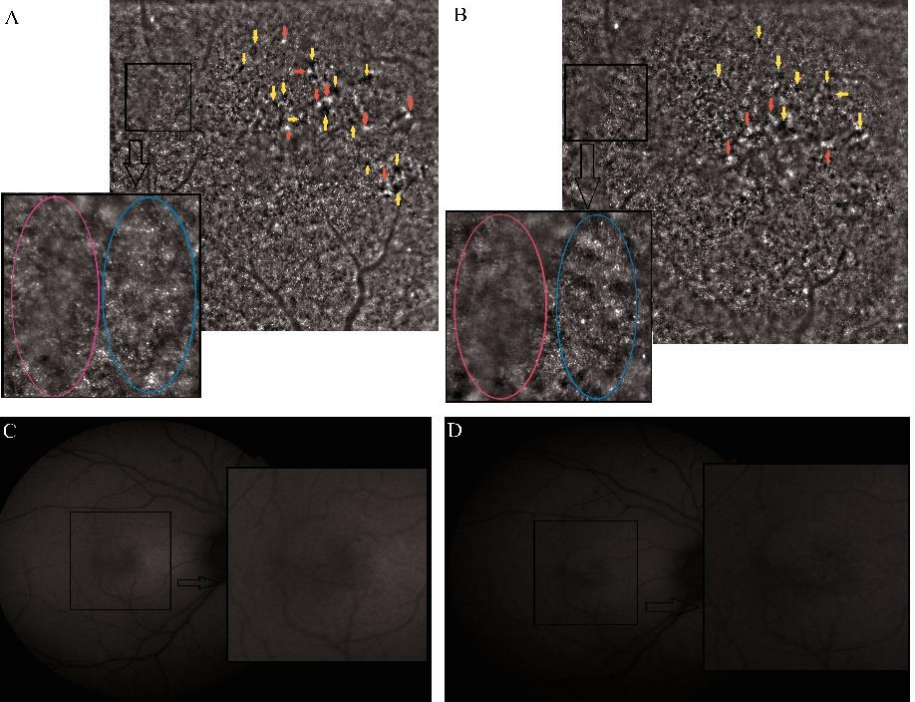
Adaptive optics high-resolution images and fundus autofluorescence (FAF) image after the laser treatment of the central serous chorioretinopathy. **A, B.** Cone mosaic at one **(A)** and at two **(B)** years after the focal LASER photocoagulation; the yellow arrows indicate black (hyporeflective) lesions; the red arrows indicate white (hyperreflective) lesions; the black arrow indicates the magnification of the cone mosaic area contained in the black square, on which normal con mosaic (blue circle) and blurred cone mosaic (pink circle) are observed; **C, D.** FAF images at one **(C)** and at two **(D)** years after the treatment; the black arrow indicates the magnification of the central area contained in the black square

Almost all hyper-AF lesions appeared white on AO cone mosaic and the hypo-AF ones appeared black [**8**]. Within a year, most black lesions on the cone mosaic decreased in size and white lesions decreased in intensity (**Fig. 2**). In the end, these changes were associated with an improvement in visual acuity.

*Case 2*

A 22-year-old female presented with the diagnosis of RP. The main symptoms were nyctalopia and loss of peripheral vision for about a year. BCVA was RE = 1.0 and left eye LE = 0.7. The funduscopic examination revealed typical clinical changes for RP: bone-spicule pigmentary changes, arteriolar attenuation, and waxy disc pallor. OCT showed a normal retinal thickness in the center of the macula, atrophy of the outer retinal layers (ORL) from the perifoveal area to the periphery in both eyes [**9**]. It was observed that the ellipsoid zone (EZ) and the interdigitation zone (IZ) on the OCT were differently impacted [**10**,**11**]. Depending on the integrity of the ENT, three areas were observed:

Area I - consisting of external limiting membrane (ELM), EZ, IZ and RPE;

Area II - consisting of ELM, EZ and RPE;

Area III - consisting of ELM and RPE.

**Fig. 3:**
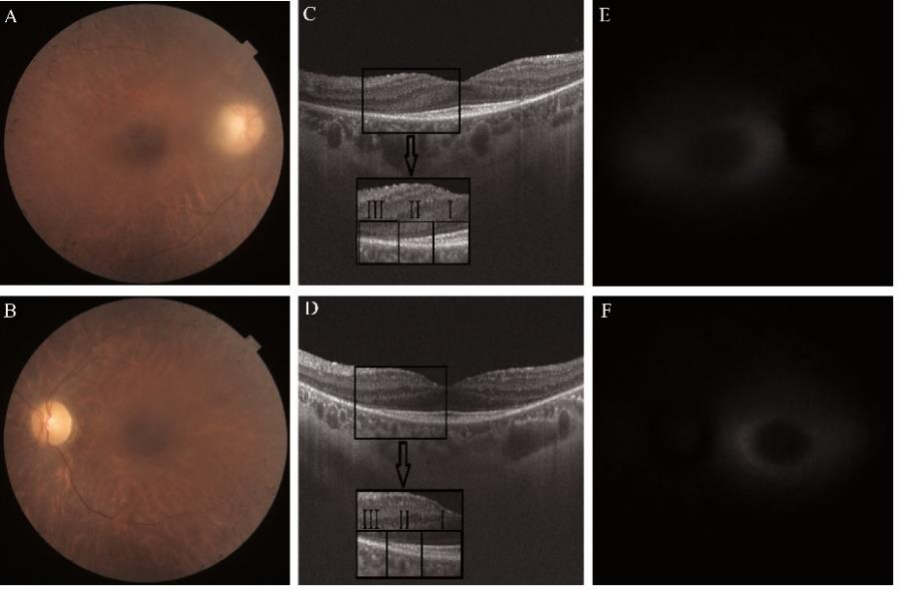
Retinitis pigmentosa. **A, B.** Color fundus images of the right and left eye. **C, D.** Optical coherence tomography scan of both eyes; the black arrow indicates the magnification of the area contained in the black square and the delimitation of the outer retinal layers in Area I, II and II. **E, F.** Fundus autofluorescence images of both eyes

The characteristic hyper-AF ring was observed in both eyes on the FAF images. Correlating these results, we observed that the hyper-AF ring corresponded to Area II of the ENT integrity (**Fig. 3**).

The AO high-resolution images were compatible with the FAF and OCT images [**12**]. Three types of cone mosaic phenotypes were observed:

- Normal cone mosaic: well-defined photoreceptor cells, located in the center of the fovea;

- Blurred cone mosaic ring: in the parafoveal area, surrounding the normal cone mosaic; a rapid deterioration of the cone mosaic from the inner to the outer edge of the ring was observed;

- Cone disappearance: lack of cone mosaic outside the blurred ring, with visible RPE.

**Fig. 4:**
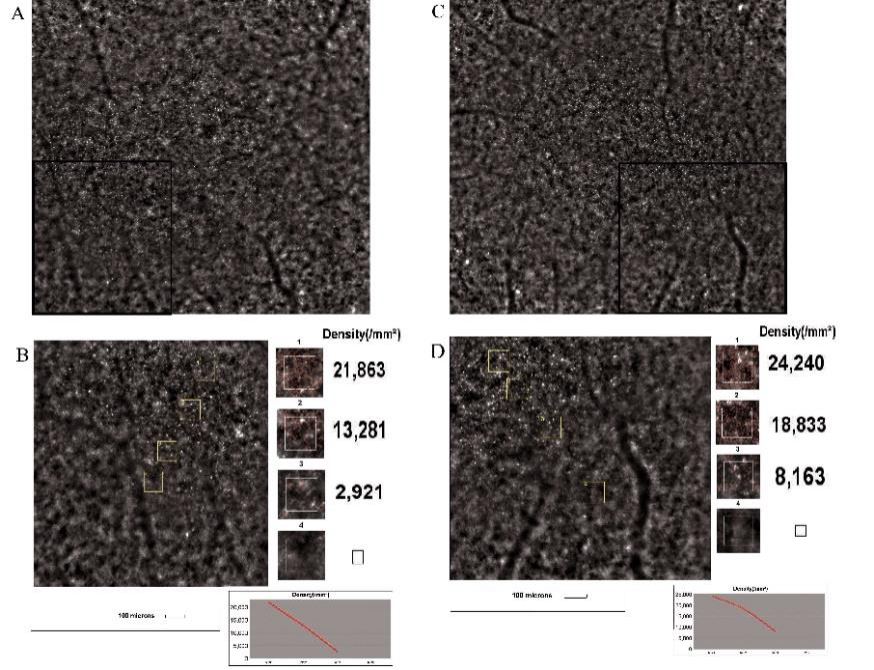
The cone mosaic and cone density results in retinitis pigmentosa. **A, B.** Cone mosaic of the right eye **(A)** and the magnification of the area contained in the black square **(B)**, with cone density measured in four points; at the bottom, the trend of cone densities can be observed. **C, D.** Cone mosaic, cone density and the trend of cone densities in the left eye

Photoreceptor density was measured in four different points from the center to the periphery. A dramatic decrease in cone density was observed and the density at the blurred ring area was impossible to determine. The highest cone density was 21.863 cones/ mm2 in the RE and 24.240 cones/ mm2 in the LE, both measured at 1º temporal to the center (**Fig. 4**).

*Case 3*

A 43-year-old male patient, diagnosed with STGD, was sent to our clinic for a second opinion. The main symptoms were binocular decrease in visual acuity, especially in the right eye, for several years, abnormalities of color vision and dark adaptation. BCVA at the presentation was RE = 0.2 and LE = 1.0. Posterior pole examination revealed: central macular atrophy that spared the fovea in the LE, “beaten bronze” appearance and yellow-white fundus flecks.

**Fig. 5:**
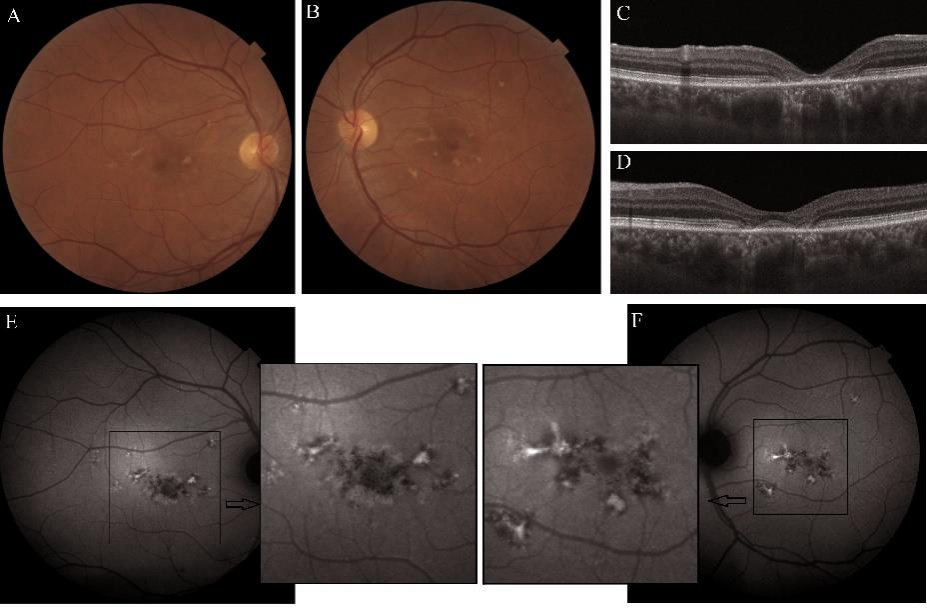
Stargardt disease. **A, B.** Color fundus images of the right (RE) and left (LE) eye. **C, D.** Optical coherence tomography scans of the RE and LE. **E, F.** Fundus autofluorescence images of the RE and LE; the black arrow indicates the magnification of area contained in the black square

The OCT showed atrophy of the central retina in both eyes and pointed out the intact fovea in the LE. FAF exam showed typical abnormalities for STGD: hyper-AF of the fundus flecks, hypo-AF of the atrophied areas with increased autofluorescence around them and sparing of the peripapillary area (**Fig. 5**). However, a small difference was observed between the FAF of the two eyes: in the RE the central hypo-AF area was surrounded by more tissue with increased autofluorescence as opposed to the left eye.

Several types of lesions could be observed on the AO high-resolution images. These are some of the cone mosaic phenotypes that were noticed:

- atrophic areas: lack of cone mosaic with visible RPE;

- periatrophic areas with increased FAF: blurred cone mosaic and less visible photoreceptors, with some areas of starry-night cone pattern;

- periatrophic areas with normal FAF: normal cone mosaic.

**Fig. 6:**
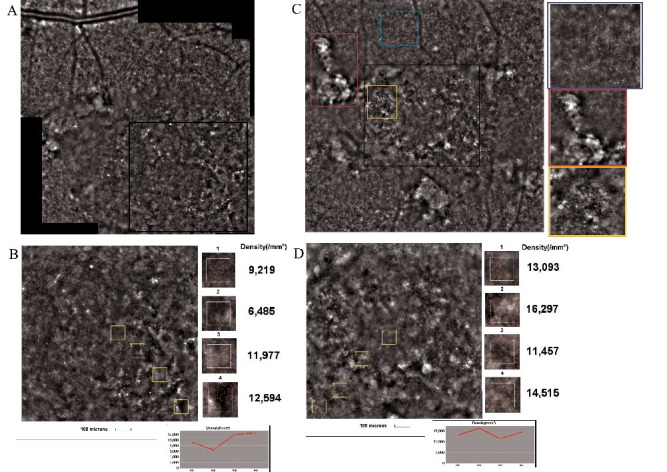
The cone mosaic and cone density results in Stargardt disease. **A, B.** Cone mosaic of the right eye **(A)** and the magnification of the area contained in the black square **(B)**, with cone density measured in four points; at the bottom, the trend of cone densities can be observed. **C, D.** Cone mosaic, cone density and the trend of cone densities in the left eye; on the right, the magnification of the cone mosaic phenotypes can be observed: normal cone mosaic (blue square), fundus flecks (red square) and lack of cone mosaic with visible retinal pigment epithelium (yellow square)

Due to the lack of fixation in the right eye, the images obtained with AO had several artifacts. In both eyes the fundus flecks appeared intensely hyperreflective, with a granular appearance, with few areas at which the cones could be observed (**Fig. 6**).

Related to the cone density, as expected, in the RE it was lower in the center than in the parafoveal area. In the LE, due to the alternation of the intact areas with the affected ones, the cone density varied. However, even in the LE it was much higher in the parafoveal region than in the fovea. The highest density of cones was 12.594 cones/ mm2 in the RE and 16.297 cones/ mm2 in the LE.

*Case 4*

**Fig. 7:**
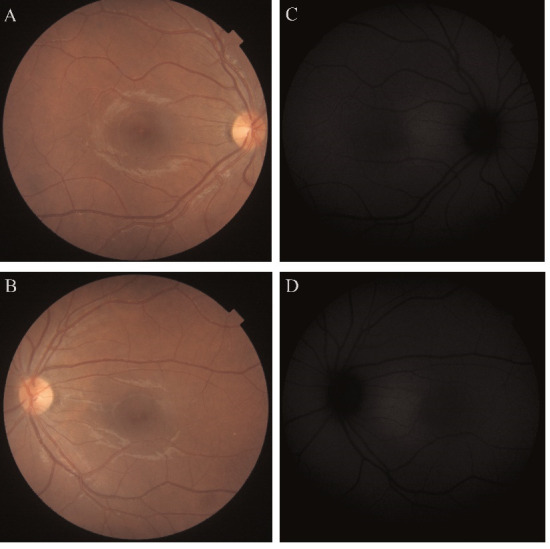
Light-induced retinopathy. **A, B.** Color fundus images of the right **(A)** and left **(B)** eye. **C, D.** Fundus autofluorescence images of both eyes

This was the case of a 17-year-old female patient, who had a central scotoma in both eyes for about a year. BCVA was 1.0 in both eyes. Nothing pathological was observed on the slit lamp biomicroscopy examination of the anterior pole. The funduscopic examination revealed the presence of yellow dot-like deposits in the fovea of both eyes (**Fig. 7**).

OCT showed the interruption of the EZ and IZ in the fovea in both eyes. FAF identified normal metabolic activity in both eyes, without autofluorescence anomalies. Considering clinical, paraclinical data and patient history, the diagnosis of light-induced retinopathy was made.

**Fig. 8:**
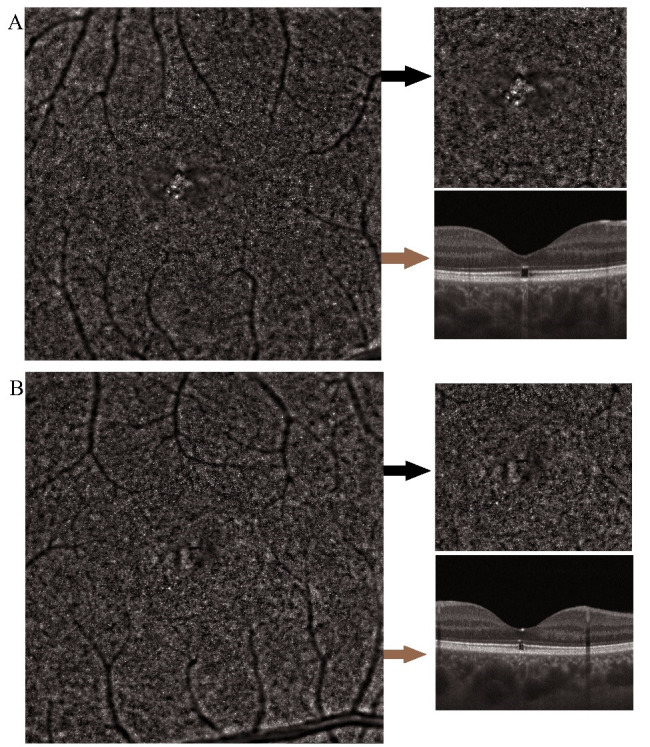
Adaptive optics high-resolution images and optical coherence tomography (OCT) in light-induced retinopathy. **A, B.** Right and left eye; the black arrow indicates the magnification of central cone mosaic; the brown arrow indicates the OCT in that area

Due to the high resolution of the images, AO was able to highlight the anatomical changes in this case in the most detailed way. In both eyes, the mosaic of cones was altered in the center of the fovea, with RPE visible in certain areas and with a blurred area around them (**Fig. 8**). The quantitative analysis showed a low cone density in the center of the fovea and at the level of the blurred cone mosaic. The density of photoreceptors increased towards the periphery, reaching normal limits in the perifoveal areas. In the RE, the cone density was 10.804 cones/ mm2 at 0.1º to the center and 25.571 cones/ mm2 at 1.12º to the center (in the LE, the results were almost the same).

## Discussion

Nowadays, the OCT is one of the most commonly used tools of examining the retinal structure. In most cases, it manages to provide the necessary information about the pathology the patient suffers from. However, as the results of this paper have shown, it is not always enough.

The presence of hypoautofluorescent lesions has already been shown to be a negative prognostic factor in CSC [**8**]. In the CSC case presented above, the leakage point was hypo-AF from the very beginning, indicating that in that area the RPE suffered the most. Furthermore, this was compatible with the pathophysiological mechanism incriminated in CSC so far [**13**].

The intense metabolic activity, represented by the autofluorescence of the macula, finally determined an improvement of the anatomical aspect on the AO high-resolution images and of the functional result. The fact that up to two years after SRF resorption metabolic changes were observed on FAF, underlined the need for a prompt, effective and fast-acting treatment in this pathology.

In the second case, the appearance of the ORL on OCT and the hyper-AF ring on FAF were typical for an incipient form of RP. Attention was drawn to the similar location of the hyper-AF ring and the blurred cone mosaic ring in Area II. If the intense metabolic activity at the level of Area II was the effect or the cause of the IZ alteration, remains to be followed in time. However, the qualitative analysis of the cone mosaic showed a normal structure of photoreceptors in the center and the quantitative analysis showed a clear decrease in cone density from the center to the blurred cone mosaic ring. 

In the case of the STGD disease, all the results were typical for this pathology. Photoreceptor density was lower in the central area compared to the periphery, and some cone mosaic phenotypes were noticed. The most remarkable in this case was the functional and anatomical difference between the right and left eye. The eye with lower visual acuity had a more intense metabolic activity associated with a more important anatomical alteration and a lower cone density. This situation was similar to that presented in the previous case with RP, and requires further studies. FAF is a very useful investigation in diagnosing the STGD, remaining to be seen if it also has a prognostic role.

In all cases, a compatibility between FAF and AO images was observed. The exception was the last case in which the high-resolution allowed the highlighting of the cellular changes, unlike FAF that did not. This was the only case in which the anatomical alteration was not associated with metabolic changes. This could be because light-induced retinopathy does not have a degenerative nature. On the other hand, FAF could not identify any changes in RPE because of the small size of the lesions.

## Conclusion

In conclusion, it could be stated that FAF and AO are very useful tools in macular pathologies examination. FAF can offer a true picture of RPE function while AO allows the view of the changes up to the cellular level.

**Conflict of Interest statement**

Authors state no conflict of interest.

**Informed Consent and Human and Animal Rights statement**

Informed consent has been obtained from all individuals included in this study.

**Authorization for the use of human subjects**

Ethical approval: The research related to human use complies with all the relevant national regulations, institutional policies, is in accordance with the tenets of the Helsinki Declaration, and has been approved by the review board of “Carol Davila” University of Medicine and Pharmacy, Bucharest, Romania.

**Acknowledgements**

None.

**Sources of Funding**

The authors have no relevant financial or non-financial interests to disclose.

**Disclosures**

None.
